# Invasive Pneumococcal Disease Epidemiology and Conjugate Vaccines in Canada, 2000-2019

**DOI:** 10.1001/jamanetworkopen.2026.6005

**Published:** 2026-04-09

**Authors:** Bernice Ramos, Nirma Khatri Vadlamudi, Alyssa R. Golden, Irene Martin, Gregory Tyrrell, Nicholas Brousseau, Manish Sadarangani

**Affiliations:** 1 Department of Microbiology and Immunology, University of British Columbia, Vancouver, Canada; 2 Department of Pediatrics, Faculty of Medicine, University of British Columbia, Vancouver, Canada; 3 Vaccine Evaluation Center, B.C. Children’s Hospital Research Institute, Vancouver, Canada; 4 National Microbiology Laboratory, Public Health Agency of Canada, Winnipeg, Canada; 5 Department of Laboratory Medicine and Pathobiology, University of Alberta, Edmonton, Canada; 6 Department of Social and Preventive Medicine, Université Laval, Quebec City, Canada; 7 Department of Pharmaceutical Outcomes & Policy, University of Florida, Gainesville

## Abstract

**Importance:**

Invasive pneumococcal disease (IPD) remains a significant burden despite implementation of pneumococcal conjugate vaccines (PCVs) in childhood immunization programs.

**Objective:**

To analyze IPD epidemiology in Canada following 7-valent pneumococcal conjugate vaccine (PCV7) and 13-valent PCV (PCV13) introductions and evaluate the potential outcomes associated with newer vaccines, such as PCV15, PCV20, and PCV21.

**Design, Setting, and Participants:**

This cross-sectional study included IPD isolates from children and adults with IPD across Canada from January 1, 2000, to December 31, 2019. Cases were identified through bacterial isolates sent to national reference laboratories. Data were analyzed from January 2022 to January 2026.

**Exposures:**

Introduction of PCV7 and PCV13.

**Main Outcomes and Measures:**

IPD incidence rates and proportion analyses were calculated for serotypes belonging to PCV7, PCV13, PCV15, PCV20, and PCV21 using Statistics Canada population estimates. PCV7 and PCV13 impact was assessed using generalized linear piecewise quasi-Poisson regression models.

**Results:**

A total of 37 921 IPD isolates were analyzed. Age data were available for 37 591 isolates (17.9% children aged <18 years; 82.1% adults aged ≥18 years), and sex data for 35 303 isolates (51.5% male; 41.6% female). Following PCV7 introduction, PCV7-specific serotype IPD rates decreased by 98.5%, from an incidence rate (IR) of 20.1 (95% CI, 17.8-22.6) in year 0 to 0.31 (95% CI, 1.0-0.5) in year 7 (*P* < .001), in children younger than 5 years (direct cohort) and 76.3%, from an IR of 2.2 (95% CI, 1.6-3.0) in year 0 to (0.5; 95% CI, 0.3-0.9) in year 7, in individuals aged 10 years or older (indirect cohort), although the difference was not statistically significant (*P* = .74). After PCV13 introduction, PCV13-only serotype IPD rates decreased by 90.6% in the direct cohort, from an IR of 10.3 (95% CI, 8.812.1) in year 0 to 1.0 (95% CI, 0.65-1.44) in year 9 (*P* = .03), and 57.1% in the indirect cohort, from an IR of 3.8 (95% CI, 3.1 to 4.6) in year 0 to 1.6 (95% CI, 1.2 to 2.1) in year 9 (*P* = .001). Vaccine serotypes 3, 4, and 19F persisted, with increases observed in adults aged 18 to 49 years and 50 to 64 years for serotypes 3 and 4, and in adults aged 65 years and older for serotype 19F. Serotype 19A cases declined after introduction of PCV13, with the highest incidence in adults aged 65 years and older. From 2015 to 2019, the potential serotype coverages by PCV15, PCV20, and PCV21 were estimated at 23% to 29%, 32% to 39%, and 36% to 49%, respectively, while nonvaccine types accounted for 45% to 52%.

**Conclusions and Relevance:**

This cross-sectional study of IPD isolates in Canada found that despite initial IPD reductions following PCVs, IPD in Canada increased, driven by emerging nonvaccine types and persistence of some vaccine serotypes. Indirect protection was limited, particularly among older adults, highlighting the need for direct adult vaccination strategies. These findings suggest that while PCV15, PCV20, and PCV21 may improve serotype coverage, continued development of broader-spectrum vaccines is essential to further reduce IPD burden.

## Key Points

**Question:**

What were the associations of the 7-valent pneumococcal conjugate vaccine (PCV7) and 13-valent PCV (PCV13) with incidence of invasive pneumococcal disease (IPD), and what future outcomes may be associated with PCV15, PCV20, and PCV21?

**Findings:**

In this cross-sectional study including 37 921 IPD isolates, PCV7 was associated with reducing IPD rates from PCV7 serotypes by 98.7% in children aged younger than 5 years and 88.4% in individuals aged 10 years and older, while PCV13 was associated with further reducing PCV13-only serotypes by 90.3% and 57.9% in these groups, respectively. Between 2015 and 2019, PCV15, PCV20, and PCV21 could have added 23% to 29%, 32% to 39%, and 36% to 49% coverage of observed IPD isolates, respectively.

**Meaning:**

These findings suggest that newer PCVs may expand protection, but persistent and nonvaccine serotype IPD incidence emphasized the need for broader-spectrum vaccines.

## Teaser

This cross-sectional study estimates incidence of invasive pneumococcal disease in Canada following introduction of pneumococcal conjugate vaccines with increasingly broad-spectrum coverage.

## Introduction

*Streptococcus pneumoniae* is the leading cause of lower respiratory infections worldwide, causing 97.9 million episodes and 505 000 deaths annually in 2021, 172 000 of which occur in children aged younger than 5 years.^1^ Invasive pneumococcal disease (IPD), where bacteria spread to sterile sites, can cause severe manifestations, such as bacteremia and meningitis.^2^ The highest incidence of IPD occurs in children aged younger than 5 years and adults aged 65 years and older.^2^ There are approximately 100 known pneumococcal serotypes^3^; the 7-valent pneumococcal conjugate vaccine (PCV7) includes serotypes 4, 6B, 9V, 14, 18C, 19F, and 23F and was approved in Canada in 2001. In 2002, the National Advisory Committee on Immunization recommended the use of PCV7 in a 3 + 1 immunization schedule (at ages 2, 4, 6, and 12 to 15 months) in all children aged younger than 5 years in most jurisdictions as part of the routine immunization program.^4^ PCV7 was implemented across Canada during 2002 to 2006, varying by province or territory. In 2010, PCV13 (added serotypes 1, 3, 5, 6A, 7F, and 19A) replaced PCV7 in most jurisdictions in a 2 + 1 schedule (at ages 2, 4, and 12 months).^5^ By 2019, 84.4% of children aged 2 years received 3 or 4 doses of PCV13.^6^

PCVs reduced the incidence rate of IPD in children, but nonvaccine serotypes (NVTs) continue to cause disease and are replacing the disease burden previously associated with serotypes covered by PCVs.^7^ Additionally, some serotypes covered by PCVs persist.^7,8,9^ The success and cost-effectiveness of PCV programs depend not only on the protection in vaccinated individuals, primarily young children, but also on providing indirect (herd) protection to unvaccinated groups, particularly adults aged 65 years and older.^10^ To improve direct protection in children and indirect protection in older adults, higher-valency vaccines are being implemented in Canada. Expanded PCV formulations, such as PCV15 (added serotypes 22F and 33F) and PCV20 (added serotypes 8, 10A, 11A, 12F, and 15B/C) have been approved for use in children in 2021 and 2022, respectively.^11^ PCV21, which contains some serotypes not present in other PCVs (serotypes 16F, 17F, 20A, 23A, 23B, 24F, 31, and 35B), was approved for use in adults aged 65 years and older in 2024.^11^ Understanding IPD epidemiology and the impact of PCVs is key to explore these strategies. This study was undertaken to describe the epidemiology of IPD in Canada from 2000 to 2019 in association with PCV7 and PCV13 use, assess protection from different PCVs, and estimate the potential outcomes associated with newer PCVs.

## Methods

Ethics approval and informed consent were not required for this cross-sectional study per the research ethics board of the Public Health Agency of Canada, as it was done as part of routine national surveillance of pneumococcal disease This study is reported following the Strengthening the Reporting of Observational Studies in Epidemiology (STROBE) reporting guideline for cross-sectional studies.

### Sample

IPD serotype data from 37 921 children and adults in Canada, excluding Quebec, from 2000 to 2019 were provided by 2 national reference laboratories. Data from 2000 to 2010 (15 376 isolates) came from the National Centre for *Streptococcus* in the Provincial Laboratory for Public Health in Edmonton, while data from 2010 to 2019 (22 545 isolates) were provided by the Public Health Agency of Canada’s National Microbiology Laboratory, which relies on passive surveillance.^12,13^ Hospitals and provincial public health laboratories voluntarily submitted IPD isolates to these libraries for serotyping during the study. Thus, this is a sample that would be representative of the serotype distribution in all IPD cases in Canada. Serotyping was performed by the Quellung reaction using pool, group, type, and factor antisera.

### Data Collection

Demographic data included age, sex, clinical source, and date of collection. Multiple isolates with the same serotype collected from different clinical sources from the same patient within 14 days were counted once, with the most invasive isolation site assigned. Meningitis-related isolates were deemed most invasive, followed by blood, and then other sterile sites. Data were aggregated by age (0-11 months, 12-23 months, 2-4 years, 5-9 years, 10-17 years, 18-49 years, 50-64 years, and ≥65 years), and regionally (British Columbia, Alberta, Manitoba, Saskatchewan, Ontario, Atlantic provinces [New Brunswick, Nova Scotia, Prince Edward Island, Newfoundland and Labrador], and the Northern territories [Northwest Territories, Yukon, and Nunavut]).

### Incidence Rate and Proportions Analysis

The entire combined dataset was used to describe the annual age-adjusted, sex-adjusted, region, vaccine serotype groups, and serotype-specific incidence rates (IRs) per 100 000 population per year analysis of IPD in Canada by calendar year from January 1, 2000, to December 31, 2019. Population estimates were taken from Statistics Canada annually on July 1, by age and sex.^14^ We estimated IPD IRs separately for 6 vaccine serotype groups defined by vaccine coverage: serotypes covered by PCV7, those added to PCV13, and non-PCV13 serotypes, which include serotypes covered by the newer higher valence vaccines PCVs (ie, PCV15, PCV20, and PCV21). For PCV15, PCV20, and PCV21, we analyzed the proportion of IPD cases attributable to serotypes included in each formulation (including serotypes covered by earlier PCVs, where applicable) from 2015 to 2019 to estimate potential population-level coverage.

To model the association of vaccine exposure with IPD IRs, the year each PCV program was introduced in a province or territory was set as year 0, with years ranging from −6 to 7 for PCV7 and −11 to 9 for PCV13 (eFigure 1 in Supplement 1), where negative years represent the period before the vaccine was implemented and positive years are after vaccine implementation. eFigure 1 in Supplement 1 illustrates the provinces and territories contributing to that data point per year. As such, year −6 for PCV7 and year −11 for PCV13 were removed to avoid data distortion due to epidemiological variations, as only the Northwest Territories and Yukon contributed data for these years, respectively.

During this time period, PCV7 and PCV13 were introduced and recommended for direct use in children aged younger than 5 years, while adults aged 65 years and older were still recommended to use 23-valent purified polysaccharide vaccine.^4,5,15^ As such, direct outcomes of the vaccine included infants and young children within the age groups targeted for vaccination (0-11 months, 12-23 months, and 2-4 years). Other age groups (10-17 years, 18-49 years, 50-64 years, and ≥65 years) were included in the indirect impact analysis. Children aged 5 to 9 years were excluded from the analysis due to the potential for incomplete protection from vaccines administered in early childhood and waning immunity.^16^

### Statistical Analysis

The associations of PCV7 and PCV13 with IPD IRs were modeled using piecewise quasi-Poisson regression to account for overdispersion. The number of cases was modeled with a log-link function, and the logarithm of the age-specific population was included as an offset to estimate IRs. A linear B-spline term (degree 1) with a single knot at year 0 was used to represent the introduction of each respective vaccine program, allowing separate linear trends before and after vaccine implementation.

Model-based estimated counts and SEs were obtained on the log scale and converted to IRs per 100 000 population by exponentiating the estimated log counts and dividing by the age-specific population (direct vs indirect cohort) before multiplying by 100 000. Approximate 95% CIs were calculated using the normal approximation on the log scale and then exponentiated, providing estimates of changes in IPD incidence associated with each PCV program. Statistical tests were two-sided, and *P* < .05 was considered statistically significant. All statistical analyses were conducted in R Studio version 4.1.1 (R Project for Statistical Computing). Data were analyzed from January 2022 to January 2026.

## Results

### Population Characteristics

A total of 37 921 IPD isolates were included, the greatest proportion of which were from Ontario (14505 isolates [38.3%]) (Table). Most isolates came from the blood (34 749 isolates [91.6%]). Age was available for 37 591 isolates; 17.9% of isolates were from children aged younger than 18 years and 82.1% were from adults aged 18 years or older. Sex data were available for 35 303 isolates (51.5% male; 41.6% female)

**Table.** Number of Cases and Proportion of Invasive Pneumococcal Disease (IPD) by Region and Clinical Characteristics During 2000 to 2019

Factor	Cases, No. (%) (N = 37 921)
Region	
Ontario	14 505 (38.3)
Alberta	8611 (22.7)
British Columbia	6969 (18.4)
Manitoba	2684 (7.1)
Saskatchewan	2635 (6.9)
Atlantic provinces^a^	2157 (5.7)
Northern territories^b^	360 (0.9)
Age group	
0-11 mo	838 (2.2)
12-23 mo	1613 (4.3)
2-4 y	2184 (5.8)
5-9 y	1073 (2.8)
10-17 y	730 (1.9)
18-49 y	9269 (24.4)
50-64 y	9483 (25.0)
≥65 y	12 401 (32.7)
Sex	
Male	19 522 (51.5)
Female	15 781 (41.6)
Vaccine serotype	
PCV7	6988 (18.4)
PCV13-only	10 733 (28.3)
PCV15-only	3973 (10.5)
PCV20-only	5907 (15.6)
PCV21-only	5966 (15.8)
NVT	4354 (11.5)
Clinical source	
Blood	34 749 (91.6)
CSF	1308 (3.4)
Lung	693 (1.8)
Bone or joint	274 (0.7)
Gastrointestinal sterile site	153 (0.4)
Central nervous system (non-CSF)	39 (0.1)
Ear, nose, and throat sterile site	43 (0.1)
Unknown	400 (0.1)
Other^c^	262 (0.7)

Abbreviations: CSF, cerebrospinal fluid; NCV, nonvaccine type; PCV7, 7-valent pneumococcal conjugate vaccine.

^a^ Atlantic provinces include Newfoundland and Labrador, Nova Scotia, New Brunswick, and Prince Edward Island.

^b^ Northern territories include Northwest Territories, Nunavut, Yukon.

^c^ Other clinical sources include eye, heart, pelvis, postmortem, renal tract sterile site, and urine.

### IPD IRs in Canada From 2000 to 2019

**Figure 1.**
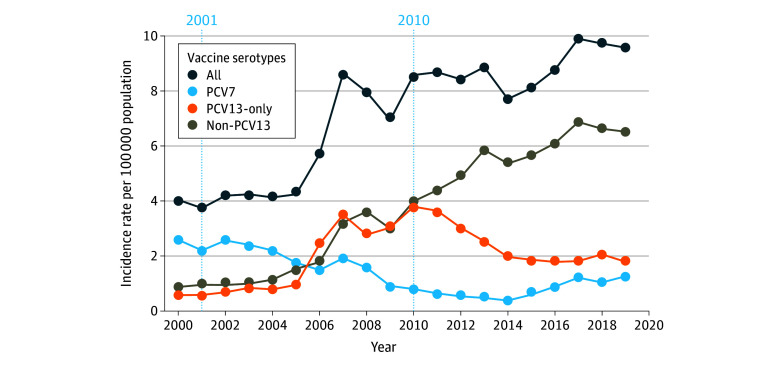
Line Graph of Age-Adjusted Invasive Pneumococcal Disease Incidence Rates in Canada Overall and by Serotype Groups Based on Vaccine Coverage PCV7 indicates pneumococcal 7-valent conjugate vaccine; PCV13, pneumococcal 13-valent conjugate vaccine.

The age-adjusted IPD IR in the general population increased from 2000 (IR, 4.0; 95% CI, 3.8-4.3) to 2019 (IR, 9.6; 95% CI, 9.2-9.9) (Figure 1). IPD IRs for each province and territory are in eFigure 2 in Supplement 1. After PCV7 introduction, PCV7-specific serotype IRs decreased from 2001 (IR, 2.2; 95% CI, 2.0-2.4) to 2014 (IR, 0.4; 95% CI 0.3–0.5), before increasing again by 2019 (IR, 1.3; 95% CI, 1.1-1.4). PCV13-only serotype IRs increased from 2005 (IR, 1.0; 95% CI, 0.8-1.1) to 2010 (IR, 3.8; 95% CI, 3.6-4.0), followed by a decrease by 2019 (IR, 1.8; 95% CI, 1.6-2.0) after PCV13 introduction. IRs of non-PCV13 serotypes increased from 4.9 (95% CI, 3.7-4.2) in 2010 to 6.5 (95% CI, 5.0-6.2) in 2019. IPD IRs by age group are presented in eFigure 3 in Supplement 1.

### Modeling Changes in IPD IRs Following PCV7 Introduction

#### Direct Cohort

**Figure 2.**
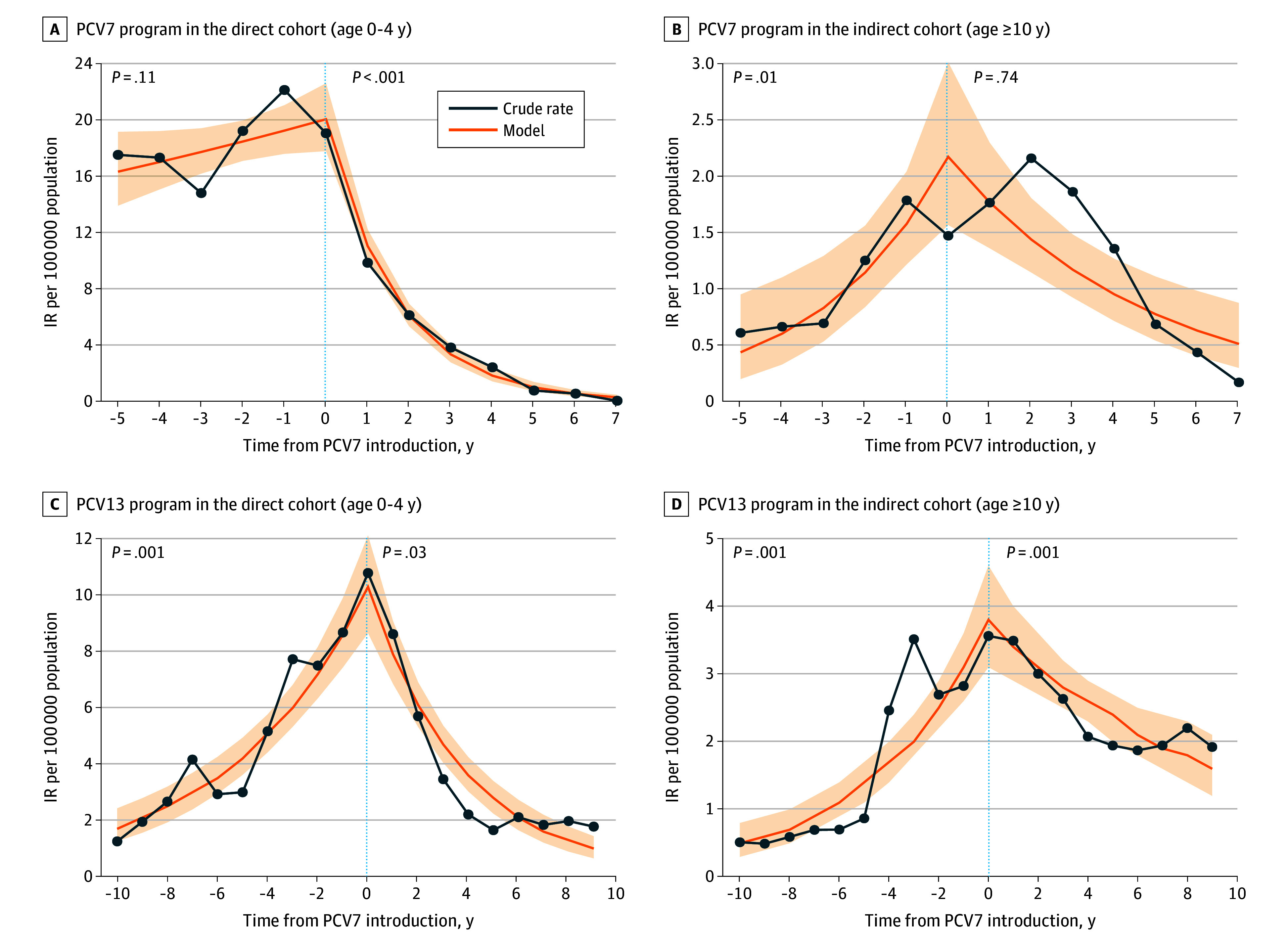
Line Graphs of Quasi-Poisson Piecewise Regression Models Showcasing the Changes in Incidence Rates (IRs) Associated With the PCV7 Program Year 0 denotes the year of pneumococcal 7-valent conjugate vaccine (PCV7) (A and B) or pneumococcal 13-valent conjugate vaccine (PCV13) (C and D) introduction. Blue dots indicate individual data points; orange line, smoothed trend; shading, 95% CI.

IPD IRs in the direct cohort (aged <5 years) due to PCV7-specific serotypes decreased by 98.5% from 20.1 (95% CI, 17.8-22.6) in year 0 to 0.31 (95% CI, 1.0-0.5) in year 7 (*P* < .001) (Figure 2A). There was some variation among provinces and territories with decreases ranging from 72.1% in the Northern Territories to 98.9% in Alberta (eFigure 4 in Supplement 1). In Ontario and the Atlantic provinces, IRs were decreasing prior to PCV7 introduction.

#### Indirect Cohort

IPD IRs in the indirect cohort (aged ≥10 years) overall for PCV7-specific serotypes decreased by 76.3% from year 0 (IR, 2.2; 95% CI, 1.6-3.0) to year 7 (IR, 0.5; 95% CI, 0.3-0.9) (*P* = .74) (Figure 2B). There was also some variation among provinces and territories with decreases ranging from 44.9% in British Columbia to 72.7% in Alberta (eFigure 4 in Supplement 1).

### Modeling Changes in IPD IRs Following PCV13 Introduction

#### Direct Cohort

Prior to PCV13 introduction, IRs for PCV13-only serotypes increased from year −10 (IR, 1.7; 95% CI, 1.3 to 2.4) to year 0 (IR, 10.3; 95% CI, 8.7 to 12.1) (*P* = .001) (Figure 2C). Similar trends were observed at the provincial and territorial level (eFigure 5 in Supplement 1). After PCV13 introduction, PCV13-only serotype IRs decreased by 90.6% from year 0 (IR, 10.3; 95% CI, 8.8 to 12.1) to year 9 (IR, 1.0; 95% CI, 0.7 to 1.4) (*P* = .03). There was variation among provinces and territories, with decreases ranging from 81.2% in Ontario to 92.3% in Alberta (eFigure 5 in Supplement 1).

#### Indirect Cohort

Prior to PCV13 introduction, IRs for PCV13-only IPD serotypes increased from year −10 (IR, 0.5; 95% CI, 0.3 to 0.8) to year 0 (IR, 3.8; 95% CI, 3.1 to 4.6) (*P* = .001) (Figure 2D) in the indirect cohort. This increase in IRs was observed at the regional level (eFigure 5 in Supplement 1). In British Columbia and Alberta, the increase coincided with higher incidences of serotype 5 observed from years −5 to −3 (eFigure 6 in Supplement 1). After PCV13 introduction, PCV13-only serotype IRs decreased by 57.1% from year 0 (IR, 3.8; 95% CI, 3.1 to 4.6) to year 9 (IR, 1.6; 95% CI, 1.2 to 2.1) (*P* = .001). There was variation among provinces and territories, with decreases ranging from 20.2% in the Atlantic provinces to 85.3% in Manitoba.

### Proportion and IRs Analysis of PCV15, PCV20, and PCV21 Vaccine Types

**Figure 3.**
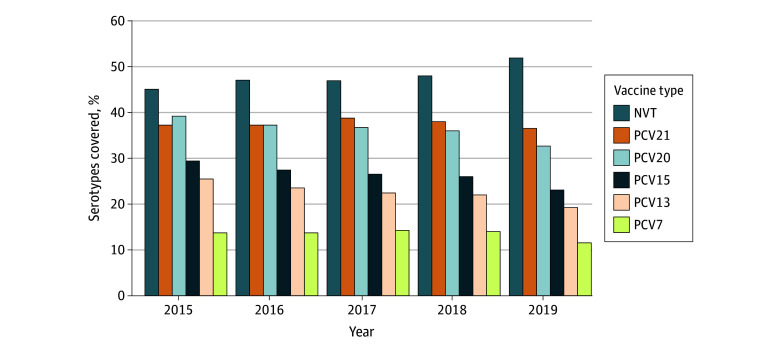
Bar Graph of Coverage of Serotypes in All Age Groups by Pneumococcal Conjugate Vaccines (PCV) and PCV21, and Nonvaccine Serotypes (NVTs) in Canada PCV7 includes 7 valents (4, 6B, 9V, 14, 18C, 19F, and 23F); PCV13, PCV7 plus 1, 3, 5, 6A, 7F, and 19A; PCV15, PCV13 plus 22F and ,33F; PCV20, PCV13 plus 8, 10A, 11A, 12F, and 15B/C; PCV21, PCV20 plus 16F, 17F, 20A, 23A, 23B, 24F, 31, and 35B. NVTs were serotypes not in any PCVs.

From 2015 to 2019, PCV15 could have covered 23% to 29% of serotypes (Figure 3). Of the PCV15-only serotypes, serotype 22F showed higher IRs than serotype 33F in both the direct and indirect cohorts (eFigure 7 in Supplement 1). PCV20 could have covered between 32% and 39% of cases from 2015 to 2019. Of the PCV20-only serotypes, serotype 15B/C had the highest IRs in the direct cohort, while serotype 8 was highest in the indirect cohort (eFigure 8 in Supplement 1). PCV21 could have covered 36% to 49% of cases between 2015 and 2019. Of the serotypes unique to PCV21, 9N had the highest IRs, followed by 23A in the indirect cohort (eFigure 9 in Supplement 1).

### Persistent Vaccine Serotypes

**Figure 4.**
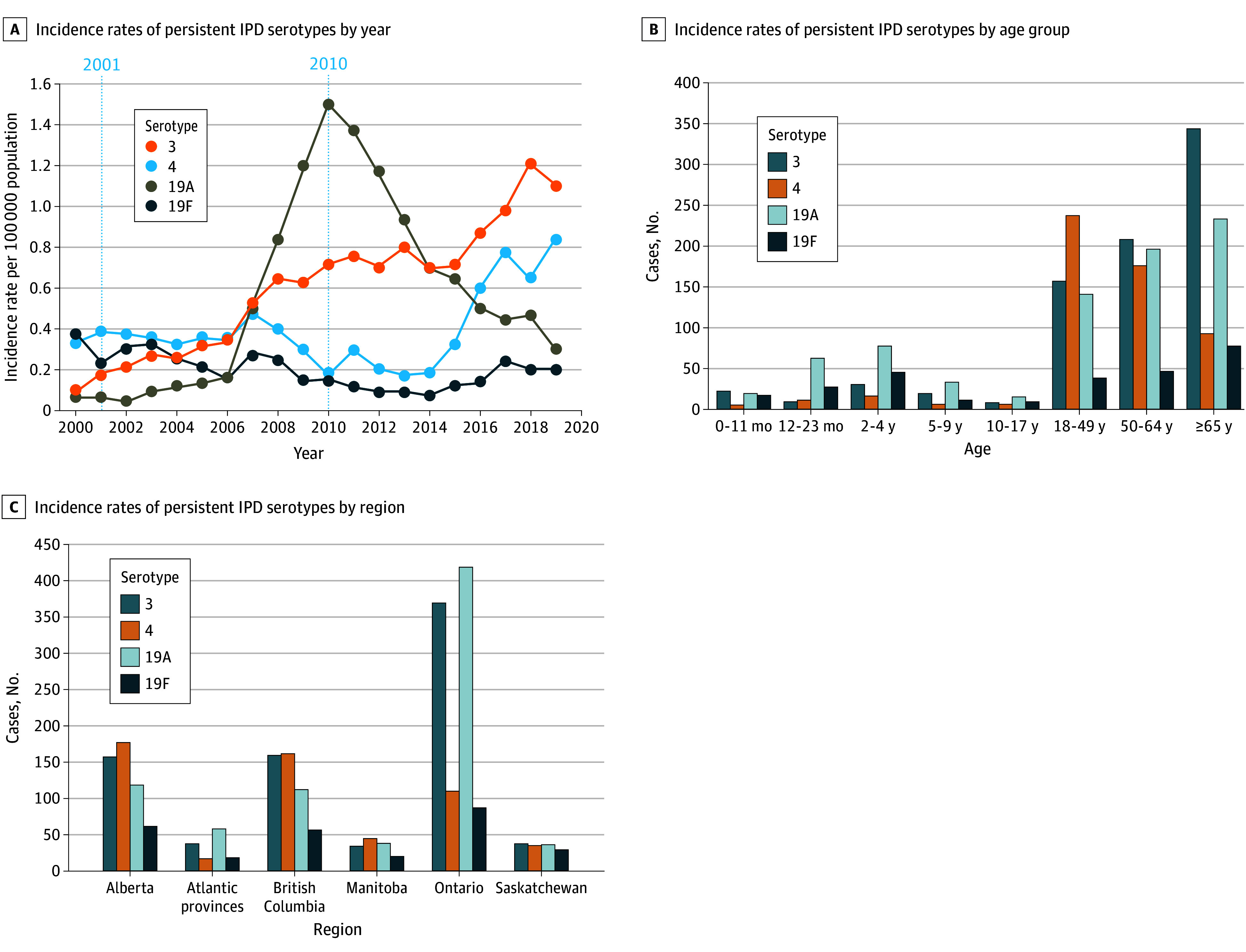
Line Graphs of Incidence Rates of Persistent Invasive Pneumococcal Disease (IPD) Serotypes in Canada and Bar Graphs of Number of Cases by Age Group and Province Northern provinces and territories were removed due to the lack of number of cases (n < 5).

Serotypes 3, 4, 19A, and 19F were the most persistent IPD serotypes despite inclusion in PCV7 and PCV13 and were frequently found in adults aged 18 years and older (Figure 4). Serotype 4 IRs initially decreased from 2001 (IR, 0.4; 95% CI, 0.3-0.5) to 2014 (IR, 0.2; 95% CI, 0.1-0.3) but increased in 2019 (IR, 0.8; 95% CI, 0.7-0.9), with the highest case numbers observed in adults aged between 18 and 49 years (236 individuals) and 50 and 64 years (175 individuals), particularly in British Columbia and Alberta (eFigure 10 and eFigure 11 in Supplement 1).

Regionally, the highest case numbers were from Ontario. By age, case numbers were highest in adults aged 65 years and older (342 individuals) and 50 to 64 years (207 individuals) between 2000 to 2019. Serotype 3 IRs increased from 2010 (IR, 0.7; 95% CI, 0.6-0.8) to 2019 (IR, 1.1; 95% CI, 1.0-1.2), increasing in all provinces except for Manitoba. In children aged younger than 5 years, serotype 3 IRs remained low and unchanged after introduction of PCV13.

Serotype 19F increased from 2014 (IR, 0.1; 95% CI, 0.04-0.1) to 2019 (IR, 0.2; 95% CI, 0.2-0.3), affecting adults aged 65 years and older, driven by increases in adults aged 50 to 64 years, particularly in British Columbia, Alberta, and Ontario. Serotype 19A had fewer cases and declined significantly after introduction of PCV13 from 2010 (IR, 1.5; 95% CI, 1.4-1.7) to 2019 (IR, 0.3; 95% CI, 0.3-0.4), with the highest incidence in adults aged 65 years and older.

## Discussion

In this cross-sectional study, we observed that IPD IRs from serotypes included in PCV7 and PCV13 were significantly reduced in Canada; however, overall IPD IRs continued to increase due to NVTs. In children aged younger than 5 years (direct cohort), IPD IRs decreased by 99% for PCV7-specific serotypes and 95.5% for PCV13-only serotypes. In individuals aged 10 years and older (indirect cohort), reductions were 77.3% for PCV7-specific serotypes and 57.9% for PCV13-only serotypes, which suggested limited indirect protection, particularly adults aged 50 years and older. From 2015 to 2019, PCV15 (covering 23%-29% of serotypes), PCV20 (covering 32%-39% of IPD serotypes), and PCV21 (covering 36%-49% of IPD serotypes) would have expanded serotype coverage. While PCV7 and PCV13 were associated with successfully reducing IPD IRs in young children, increasing NVTs and persistent serotypes covered by PCVs in adults underscore the need for updated vaccines and potential direct vaccination in older populations.

Following PCV7 introduction, there was a significant reduction in constituent serotype IRs in the direct cohort. Regionally, IRs were already decreasing in provinces such as Manitoba, Saskatchewan, Ontario, and the Atlantic provinces in the direct cohort, but this was not consistently reflected in the indirect cohort. This may be due to changes in surveillance methods. In the lead-up to a vaccine program, there are often changes in disease awareness and surveillance that are not always formally captured. It is also possible that such decreases could be due to private vaccination. However, after PCV7 introduction, IRs for IPD with PCV13-only serotypes increased in both cohorts, indicating that the IPD burden was being replaced by what were at that time NVTs. In Western Canada, particularly British Columbia and Alberta, an increase in IPD was observed in the indirect cohort between years −5 and −3, likely due to an outbreak of serotype 5 in impoverished urban areas.^9^

The significant decrease of PCV13-only serotype IRs in the direct cohort following PCV13 introduction aligned with data from other countries that implemented PCV13 as a 2 + 1 schedule. A European multicenter observational study in children found that countries that implemented a PCV13 2 + 1 schedule (ie, Denmark, England, France, Ireland, Norway, and Scotland) had a vaccine effectiveness of 76% if children received 2 doses before age 12 months.^17^ In Uruguay, PCV13-only serotype IPD IRs in children aged younger than 5 years old decreased by 83.9%.^18^

In the indirect cohort, PCV13-only IPD IRs decreased by only 58% after PCV13 introduction and remained relatively unchanged between years 4 and 9, suggesting limited indirect protection, particularly in adults aged 50 years and older. Data on PCV13 indirect effectiveness were conflicting. The Pneumococcal Serotype Replacement and Distribution Estimation Project, which includes surveillance from 76 countries (32 using PCV13 in 3 + 0, 3 + 1, or 2 + 1 schedules), found that during the late PCV13 period, cases of PCV13-only serotypes causing pneumococcal meningitis were low, affecting 15.3% of children aged younger than 5 years and 25.8% of adults.^19,20^ The limited reduction in adults observed was likely attributed to persistent serotypes 3 and 19A.^20^ A European study on PCV13 effectiveness in pediatric national immunization programs found low rates of PCV13-only IPD serotypes (28.1%) in older adults, although serotypes 3 and 19A persisted.^21^

The proportion of IPD cases due to the 2 serotypes added to PCV15 increased in Canada. Serotype 22F IRs were higher than 33F in the direct cohort. Similar trends were reported in England and Spain in children aged 0 to 17 years.^22,23^ The US, using a 3 + 1 schedule, reported IPD cases from serotypes 22F and 33F increasing from 5% and 7% in 2009 to 19% and 12% in 2017, respectively.^24^ Serotype 22F IRs were also higher than 33F in the indirect cohort. In England, these serotypes increased significantly in adults aged 18 years and older, which began following PCV7 introduction.^22^ In France, a 17-year study found serotype 22F among the most common serotypes in adults aged 65 years and older, increasing from 6.8% (2011-2014) to 8.1% (2015-2017), while 33F was less prevalent.^25^

The proportion of IPD cases due to the 5 serotypes added to PCV15 also increased in Canada. In the direct cohort, serotype 15B had the highest IRs, while serotype 8 was highest in the indirect cohort. In France during the late PCV13 period (2015-2017), serotype 24F (24.4%) was most common in children, followed by 15B/C (8.1%), while serotype 8 caused only 4% of cases in children younger than 2 years.^25^ Among adults aged 65 years and older, serotype 8 (6.9%) was the leading cause of IPD.^25^ In Belgium, serotype 8 caused 3.6% of cases in children aged younger than 2 years and 30% in adults aged 65 years and older, while 15B contributed to 2.2% and 2%, respectively.^26^ In some Nordic countries, serotype 8 was the leading cause of IPD in adults aged 65 years and older in 2019.^27^ In contrast, a study in Asia reported high numbers of serotype 11A and 23A from 2015 to 2017 in both children and adults.^28^ These variations highlighted that while expanding PCV valency remains beneficial, regional serotype distribution impacts global vaccine efficacy. Indirect protection also depends on high childhood vaccine coverage, with countries achieving high coverage reporting lower IPD in adults aged 65 years and older.^10^ In Canada, the 85% vaccine uptake in children in 2021 suggests PCV15, PCV20, and PCV21 could still provide adequate indirect protection.^11^ Direct adult vaccination, particularly adults aged 65 years and older and those aged 18 to 64 years with chronic medical conditions, should be strongly recommended, as the reported vaccine uptake for these groups in 2021 was 55% and 26%, respectively.^29^

Serotypes 3, 4, and 19F persisted in causing IPD. We observed an increase in serotype 3 IPD. Serotype 3 IPD IRs remained low and unchanged in children since PCV13 introduction but increased in adults, suggesting limited indirect protection. Many countries also reported high serotype 3 IPD IRs both in children and adults during the late PCV13 period.^25,27,28^ PCV13 effectiveness against serotype 3 has been found to wane over time, providing no lasting protection.^30^ Some data comparing PCV15 and PCV20 to PCV13 using a 2 + 1 schedule suggest that PCV15 may provide better protection in infants than PCV13 and PCV20 against serotype 3.^31^ Serotype 4 IPD was also increasing in adults, likely due to outbreaks among persons experiencing homelessness in Alberta and British Columbia.^9,32^ Similar outbreaks were reported in persons experiencing homelessness in Colorado, California, and New Mexico between 2015 and 2018.^33^ Although serotype 19A and 19F IPD IRs remained relatively low in Canada, 19F had slightly increased. These serotypes, associated with antibiotic resistance, continue to cause IPD in other countries using PCV13.^7,28^

### Strength and Limitations

A key strength of this study is the use of piecewise quasi-Poisson regression models, which accounted for varying PCV7 and PCV13 implementation across provinces and territories. This approach enabled a nuanced analysis of IRs before and after vaccine introduction, providing a clearer understanding of PCV impact without relying on individual vaccine status. It also allowed assessment of direct and indirect effects through national immunization programs and population-level IPD IRs.

This study has some limitations. It relies on passive surveillance, with voluntary isolate submissions to National Reference Centers. Between 2013 and 2019, 74.6% to 96.8% of cases were submitted to the National Microbiology Laboratory compared with those reported in the Canadian Notifiable Disease Surveillance System.^34^ Also, Quebec IPD isolates were excluded, potentially introducing information bias; therefore, findings may not fully reflect true IPD IRs in Canada.

## Conclusions

The findings of this cross-sectional study suggest PCVs remained highly effective at reducing IPD vaccine-type IRs in individuals who were directly immunized. The National Advisory Committee on Immunization in Canada recommends PCV15 or PCV20 should be used for routine immunization of healthy children aged younger than 5 years, while PCV20 or PCV21 is recommended for adults aged 65 years and older.^11^ Hopefully, the use of PCV15, PCV20, and PCV21 will decrease IPD IRs due to recently circulating serotypes across all age groups. Improving overall PCV effectiveness will require vaccinating both pediatric and adult populations, especially considering the limited indirect protection in adults. Furthermore, the continuous threat of emerging NVTs causing IPD remains a significant challenge, highlighting the need for a new generation of broad-coverage vaccines.

## Notes

Supplement 1. eFigure 1. Introduction of PCV7 and PCV13 in different provinces and territories eFigure 2. Age-adjusted IPD incidence rates between 2000 to 2019 in by vaccine type and region. eFigure 3. IPD incidence rates between 2000 to 2019 in Canada by vaccine type and age-group. eFigure 4. Quasi-Poisson piecewise regression models showcasing the changes in incidence rates associated with the PCV7 program by region eFigure 5. Quasi-Poisson piecewise regression models showcasing the changes in incidence rates associated with the PCV13 program by region eFigure 6. Breakout of serotypes 5 in Alberta and British Columbia from year -5 to year -3 during the observation of the PCV13 program in the indirect population eFigure 7. Incidence rates of PCV15/non-PCV13 serotypes in the direct and indirect cohorts eFigure 8. Incidence rates of PCV20/non-PCV13 serotypes in the direct and indirect cohorts eFigure 9. Incidence rates of PCV21/non-PCV20 serotypes in the direct and indirect cohorts eFigure 10. Incidence rates of persistent serotypes 3, 4, 19A, and 19F by age group eFigure 11. Incidence rates of persistent serotypes 3, 4, 19A, and 19F by province

Supplement 2. Data Sharing Statement

## References

[1] Bender RG, Sirota SB, Swetschinski LR, ; GBD 2021 Lower Respiratory Infections and Antimicrobial Resistance Collaborators. Global, regional, and national incidence and mortality burden of non–COVID-19 lower respiratory infections and aetiologies, 1990-2021: a systematic analysis from the Global Burden of Disease Study 2021. Lancet Infect Dis. 2024;24(9):974-1002. doi:10.1016/S1473-3099(24)00176-2 38636536 PMC11339187

[2] Henriques-Normark B, Tuomanen EI. The pneumococcus: epidemiology, microbiology, and pathogenesis. Cold Spring Harb Perspect Med. 2013;3(7):a010215-a010215. doi:10.1101/cshperspect.a010215 23818515 PMC3685878

[3] Ganaie F, Saad JS, McGee L, . A new pneumococcal capsule type, 10D, is the 100th serotype and has a large cps fragment from an oral streptococcus. mBio. 2020;11(3):e00937-e20. doi:10.1128/mBio.00937-20 32430472 PMC7240158

[4] National Advisory Committee on Immunization. An Advisory Committee Statement (ACS). National Advisory Committee on Immunization (NACI). Statement on recommended use of pneumococcal conjugate vaccine. Can Commun Dis Rep. 2002;28(ACS-2):1-32. 12728645

[5] Desai S, McGeer A, Quach-Thanh C, Elliott D; approved by NACI. Update on the use of conjugate pneumococcal vaccines in childhood: an advisory committee statement (ACS) National Advisory Committee on Immunization (NACI). Can Commun Dis Rep. 2010;36(ACS-12):1-21. doi:10.14745/ccdr.v36i00a12 31697280 PMC6802447

[6] Government of Canada. Vaccination coverage goals and vaccine preventable disease reduction targets by 2025. Modified August 16, 2022. Accessed February 26, 2026. https://www.canada.ca/en/public-health/services/immunization-vaccine-priorities/national-immunization-strategy/vaccination-coverage-goals-vaccine-preventable-diseases-reduction-targets-2025.html

[7] Kandasamy R, Voysey M, Collins S, . Persistent circulation of vaccine serotypes and serotype replacement after 5 years of infant immunization with 13-valent pneumococcal conjugate vaccine in the United Kingdom. J Infect Dis. 2020;221(8):1361-1370. 31004136 10.1093/infdis/jiz178

[8] Gounder PP, Bruden D, Rudolph K, . Re-emergence of pneumococcal colonization by vaccine serotype 19F in persons aged ≥5 years after 13-valent pneumococcal conjugate vaccine introduction—Alaska, 2008-2013. Vaccine. 2018;36(5):691-697. doi:10.1016/j.vaccine.2017.12.035 29279284

[9] Tyrrell GJ, Lovgren M, Ibrahim Q, . Epidemic of invasive pneumococcal disease, western Canada, 2005-2009. Emerg Infect Dis. 2012;18(5):733-740. doi:10.3201/eid1805.110235 22515944 PMC3358065

[10] Tsaban G, Ben-Shimol S. Indirect (herd) protection, following pneumococcal conjugated vaccines introduction: a systematic review of the literature. Vaccine. 2017;35(22):2882-2891. doi:10.1016/j.vaccine.2017.04.032 28449971

[11] Public Health Agency of Canada. Pneumococcal vaccines: Canadian immunization guide. Modified September 10, 2024. Accessed January 6, 2025. https://www.canada.ca/en/public-health/services/publications/healthy-living/canadian-immunization-guide-part-4-active-vaccines/page-16-pneumococcal-vaccine.html

[12] Public Health Agency of Canada. National laboratory surveillance of invasive streptococcal disease in Canada—annual summary 2014. Accessed May 21, 2024. https://www.canada.ca/content/dam/canada/health-canada/migration/healthy-canadians/publications/drugs-products-medicaments-produits/2014-streptococcus/alt/surveillance-streptococca-eng.pdf

[13] Public Health Agency of Canada. National laboratory surveillance of invasive streptococcal disease in Canada—annual summary 2019. Accessed May 21, 2024. https://www.canada.ca/en/public-health/services/publications/drugs-health-products/national-laboratory-surveillance-invasive-streptococcal-disease-canada-annual-summary-2019.html

[14] Statistics Canada. Table 18-10-0005-01 population estimates on July1, by age and gender. Accessed January 6, 2025. https://www150.statcan.gc.ca/t1/tbl1/en/tv.action?pid=1710000501

[15] Quach-Thanh C, Thomas MH; approved by NACI. Statement on the Use of Conjugate Pneumococcal Vaccine - 13 Valent in Adults (Pneu-C-13): An Advisory Committee Statement (ACS) National Advisory Committee on Immunization (NACI). Can Commun Dis Rep. 2013;39(ACS-5):1-52. doi:10.14745/ccdr.v39i00a05 31682649 PMC6802426

[16] Jayasinghe S, Chiu C, Quinn H, Menzies R, Gilmour R, McIntyre P. Effectiveness of 7- and 13-valent pneumococcal conjugate vaccines in a schedule without a booster dose: a 10-year observational study. Clin Infect Dis. 2018;67(3):367-374. doi:10.1093/cid/ciy129 29471432

[17] Savulescu C, Krizova P, Valentiner-Branth P, ; SpIDnet VE study group. Effectiveness of 10 and 13-valent pneumococcal conjugate vaccines against invasive pneumococcal disease in European children: SPIDNET observational multicentre study. Vaccine. 2022;40(29):3963-3974. doi:10.1016/j.vaccine.2022.05.011 35637067

[18] García Gabarrot G, López Vega M, Pérez Giffoni G, ; Uruguayan SIREVA II Group. Effect of pneumococcal conjugate vaccination in Uruguay, a middle-income country. PLoS One. 2014;9(11):e112337. doi:10.1371/journal.pone.0112337 25375647 PMC4223029

[19] Deloria Knoll M, Bennett JC, Garcia Quesada M, ; The Pserenade Team. Global landscape review of serotype-specific invasive pneumococcal disease surveillance among countries using PCV10/13: the Pneumococcal Serotype Replacement and Distribution Estimation (PSERENADE) Project. Microorganisms. 2021;9(4):742. doi:10.3390/microorganisms9040742 33918127 PMC8066045

[20] Garcia Quesada M, Yang Y, Bennett JC, ; The Pserenade Team. Serotype distribution of remaining pneumococcal meningitis in the mature PCV10/13 period: findings from the PSERENADE Project. Microorganisms. 2021;9(4):738. doi:10.3390/microorganisms9040738 33916227 PMC8066874

[21] Grant LR, Slack MPE, Theilacker C, . Distribution of serotypes causing invasive pneumococcal disease in older adults from high-income countries and impact of pediatric and adult vaccination policies. Vaccine. 2023;41(38):5662-5669. doi:10.1016/j.vaccine.2023.08.001 37544825

[22] Amin-Chowdhury Z, Groves N, Sheppard CL, . Invasive pneumococcal disease due to 22F and 33F in England: a tail of two serotypes. Vaccine. 2021;39(14):1997-2004. doi:10.1016/j.vaccine.2021.02.026 33715901

[23] Sempere J, de Miguel S, González-Camacho F, Yuste J, Domenech M. Clinical relevance and molecular pathogenesis of the emerging serotypes 22F and 33F of *Streptococcus pneumoniae* in Spain. Front Microbiol. 2020;11:309. doi:10.3389/fmicb.2020.00309 32174903 PMC7056674

[24] Hu T, Weiss T, Owusu-Edusei K, Petigara T. Health and economic burden associated with 15-valent pneumococcal conjugate vaccine serotypes in children in the United States. J Med Econ. 2020;23(12):1653-1660. doi:10.1080/13696998.2020.1840216 33084447

[25] Ouldali N, Varon E, Levy C, . Invasive pneumococcal disease incidence in children and adults in France during the pneumococcal conjugate vaccine era: an interrupted time-series analysis of data from a 17-year national prospective surveillance study. Lancet Infect Dis. 2021;21(1):137-147. doi:10.1016/S1473-3099(20)30165-1 32702302

[26] Janssens E, Flamaing J, Vandermeulen C, Peetermans WE, Desmet S, De Munter P. The 20-valent pneumococcal conjugate vaccine (PCV20): expected added value. Acta Clin Belg. 2023;78(1):78-86. doi:10.1080/17843286.2022.2039865 35171752

[27] Palmborg A, Skovdal M, Molden T, Åhman H, Chen L, Banefelt J. Invasive pneumococcal disease among the elderly in the later era of paediatric pneumococcal conjugate vaccination—a longitudinal study over 10 years based on public surveillance data in the Nordics. PLoS One. 2023;18(6):e0287378. doi:10.1371/journal.pone.0287378 37363884 PMC10292715

[28] Kim SH, Chung DR, Song JH, ; Asian Network for Surveillance of Resistant Pathogens (ANSORP). Changes in serotype distribution and antimicrobial resistance of Streptococcus pneumoniae isolates from adult patients in Asia: emergence of drug-resistant non-vaccine serotypes. Vaccine. 2020;38(38):6065-6073. doi:10.1016/j.vaccine.2019.09.065 31590932

[29] Public Health Agency of Canada. Vaccine uptake in Canadian adults: highlights from the 2020-2021 Seasonal Influenza Vaccination Coverage Survey. Published online 2022. Accessed June 13, 2025. https://www.canada.ca/en/public-health/services/immunization-vaccines/vaccination-coverage/highlights-2020-2021-seasonal-influenza-survey/full-report.html

[30] Deceuninck G, Brousseau N, Lefebvre B, . Effectiveness of thirteen-valent pneumococcal conjugate vaccine to prevent serotype 3 invasive pneumococcal disease in Quebec in children, Canada. Vaccine. 2023;41(38):5486-5489. doi:10.1016/j.vaccine.2023.07.049 37524629

[31] De Wals P. PCV13, PCV15 or PCV20: which vaccine is best for children in terms of immunogenicity? Can Commun Dis Rep. 2024;50(1-2):35-39. doi:10.14745/ccdr.v50i12a04 38655244 PMC11037880

[32] Kellner JD, Ricketson LJ, Demczuk WHB, . Whole-genome analysis of *Streptococcus pneumoniae* serotype 4 causing outbreak of invasive pneumococcal disease, Alberta, Canada. Emerg Infect Dis. 2021;27(7):1867-1875. doi:10.3201/eid2707.204403 34152965 PMC8237880

[33] Beall B, Walker H, Tran T, . Upsurge of conjugate vaccine serotype 4 invasive pneumococcal disease clusters among adults experiencing homelessness in California, Colorado, and New Mexico. J Infect Dis. 2021;223(7):1241-1249. doi:10.1093/infdis/jiaa501 32798216 PMC8108119

[34] Government of Canada. Publications: diseases and conditions. Accessed June 13, 2025. https://www.canada.ca/en/services/health/publications/diseases-conditions.html

