# Supplementary material for: Invasive Pneumococcal Disease Epidemiology and Conjugate Vaccines in Canada, 2000-2019
Source: JAMA Netw Open. 2026 Apr 9;9(4):e266005. doi: 10.1001/jamanetworkopen.2026.6005 (PMC13067013; doi:10.1001/jamanetworkopen.2026.6005)
Supplement: Supplement 1. — eFigure 1. Introduction of PCV7 and PCV13 in different provinces and territories eFigure 2. Age-adjusted IPD incidence rates between 2000 to 2019 in by vaccine type and region. eFigure 3. IPD incidence rates between 2000 to 2019 in Canada by vaccine type and age-group. eFigure 4. Quasi-Poisson piecewise regression models showcasing the changes in incidence rates associated with the PCV7 program by region eFigure 5. Quasi-Poisson piecewise regression models showcasing the changes in incidence rates associated with the PCV13 program by region eFigure 6. Breakout of serotypes 5 in Alberta and British Columbia from year -5 to year -3 during the observation of the PCV13 program in the indirect population eFigure 7. Incidence rates of PCV15/non-PCV13 serotypes in the direct and indirect cohorts eFigure 8. Incidence rates of PCV20/non-PCV13 serotypes in the direct and indirect cohorts eFigure 9. Incidence rates of PCV21/non-PCV20 serotypes in the direct and indirect cohorts eFigure 10. Incidence rates of persistent serotypes 3, 4, 19A, and 19F by age group eFigure 11. Incidence rates of persistent serotypes 3, 4, 19A, and 19F by province [file jamanetwopen-e266005-s001.pdf]

Supplemental Online Content

Ramos B, Vadlamudi NK, Golden AR, et al; the Canadian Immunization Research Network. Invasive pneumococcal disease epidemiology and conjugate vaccines in Canada, 2000-2019. *JAMA Netw Open*. 2026;9(4):e266005. doi:10.1001/jamanetworkopen.2026.6005

eFigure 1. Introduction of PCV7 and PCV13 in different provinces and territories

eFigure 2. Age-adjusted IPD incidence rates between 2000 to 2019 in by vaccine type and region.

eFigure 3. IPD incidence rates between 2000 to 2019 in Canada by vaccine type and age-group.

eFigure 4. Quasi-Poisson piecewise regression models showcasing the changes in incidence rates associated with the PCV7 program by region

eFigure 5. Quasi-Poisson piecewise regression models showcasing the changes in incidence rates associated with the PCV13 program by region

eFigure 6. Breakout of serotypes 5 in Alberta and British Columbia from year -5 to year -3 during the observation of the PCV13 program in the indirect population

eFigure 7. Incidence rates of PCV15/non-PCV13 serotypes in the direct and indirect cohorts

eFigure 8. Incidence rates of PCV20/non-PCV13 serotypes in the direct and indirect cohorts

eFigure 9. Incidence rates of PCV21/non-PCV20 serotypes in the direct and indirect cohorts

eFigure 10. Incidence rates of persistent serotypes 3, 4, 19A, and 19F by age group

eFigure 11. Incidence rates of persistent serotypes 3, 4, 19A, and 19F by province

This supplemental material has been provided by the authors to give readers additional information about their work.

© 2026 Ramos B et al. *JAMA Network Open*.

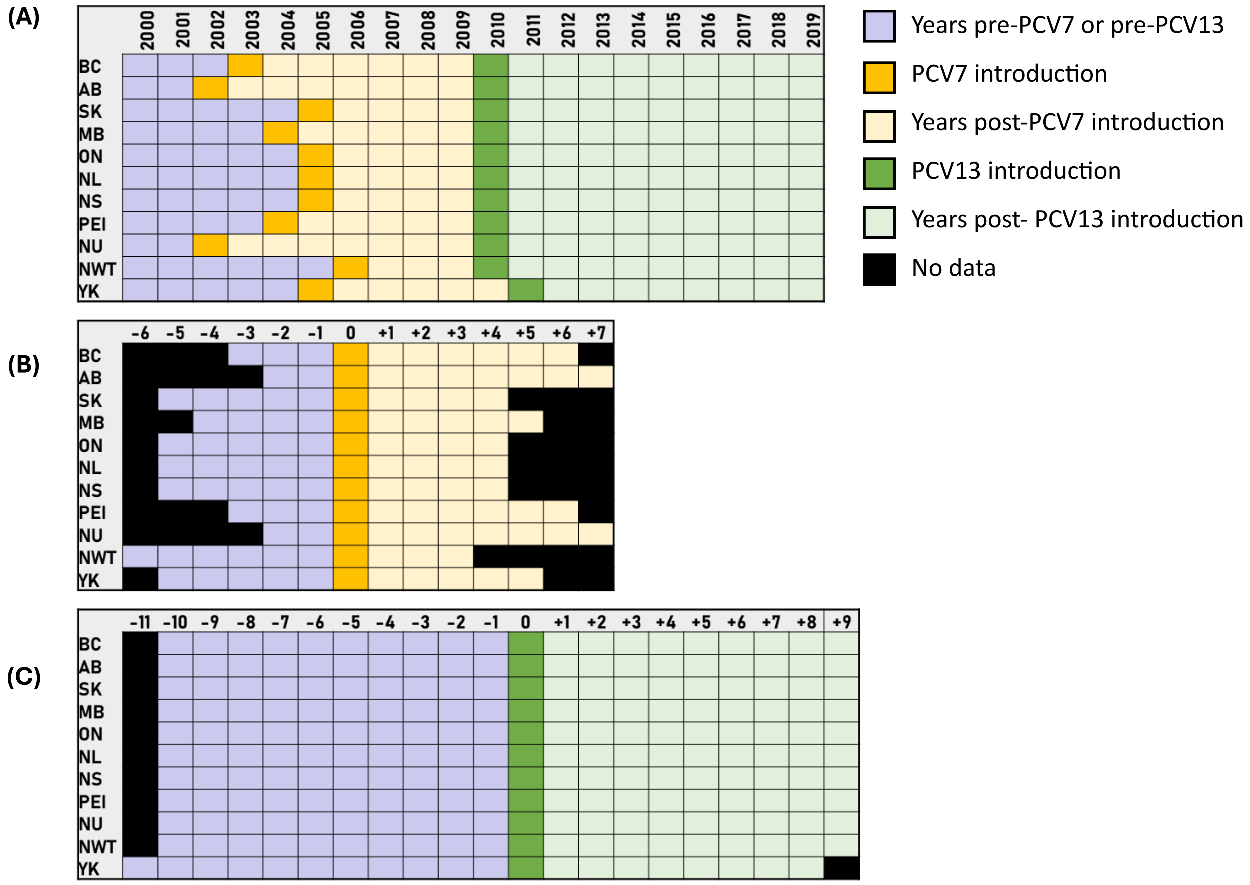

Supplementary Figure 1. Introduction of PCV7 and PCV13 in different provinces and territories where (A) is the actual years, (B) are years adjusted for PCV7 observation for PCV7 serotypes and (C) are years adjusted for PCV13 observation for PCV13/non-PCV7 serotypes that were used for the Poisson-piecewise regression models.

© 2026 Ramos B et al. *JAMA Network Open*.

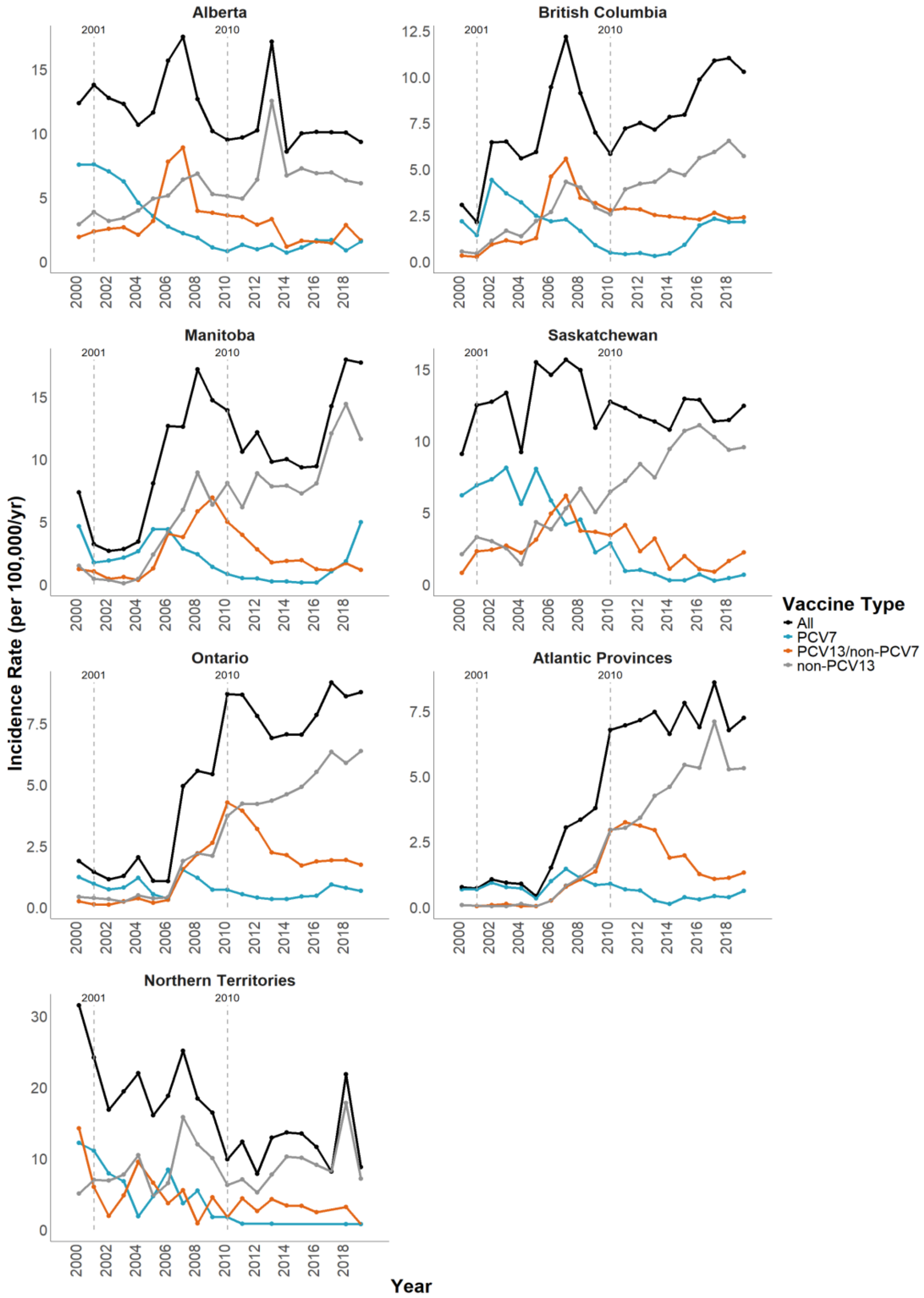

Supplementary Figure 2. Age-adjusted IPD incidence rates between 2000 to 2019 in by vaccine type and region.

© 2026 Ramos B et al. *JAMA Network Open*.

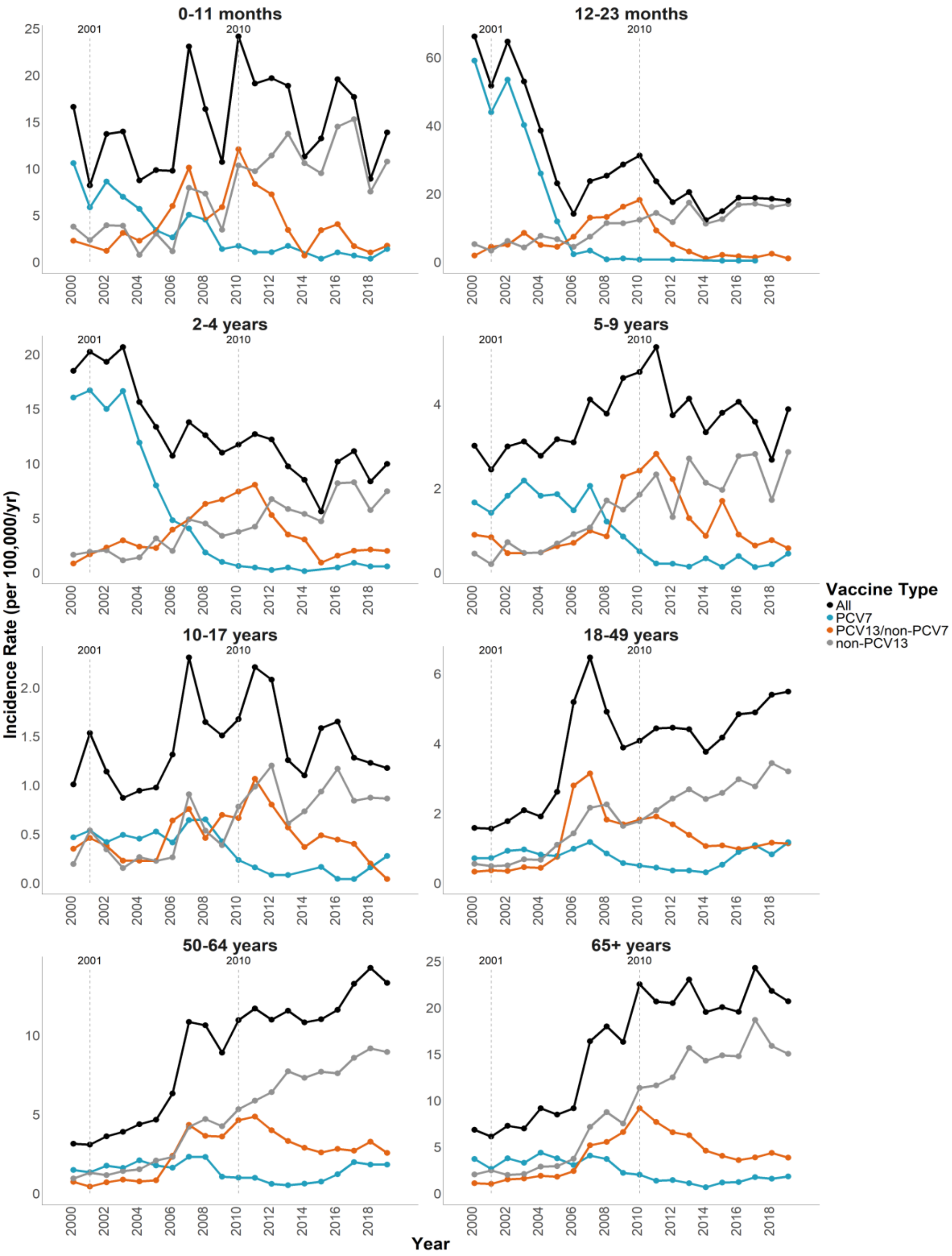

Supplementary Figure 3. IPD incidence rates between 2000 to 2019 in Canada by vaccine type and age-group.

© 2026 Ramos B et al. *JAMA Network Open*.

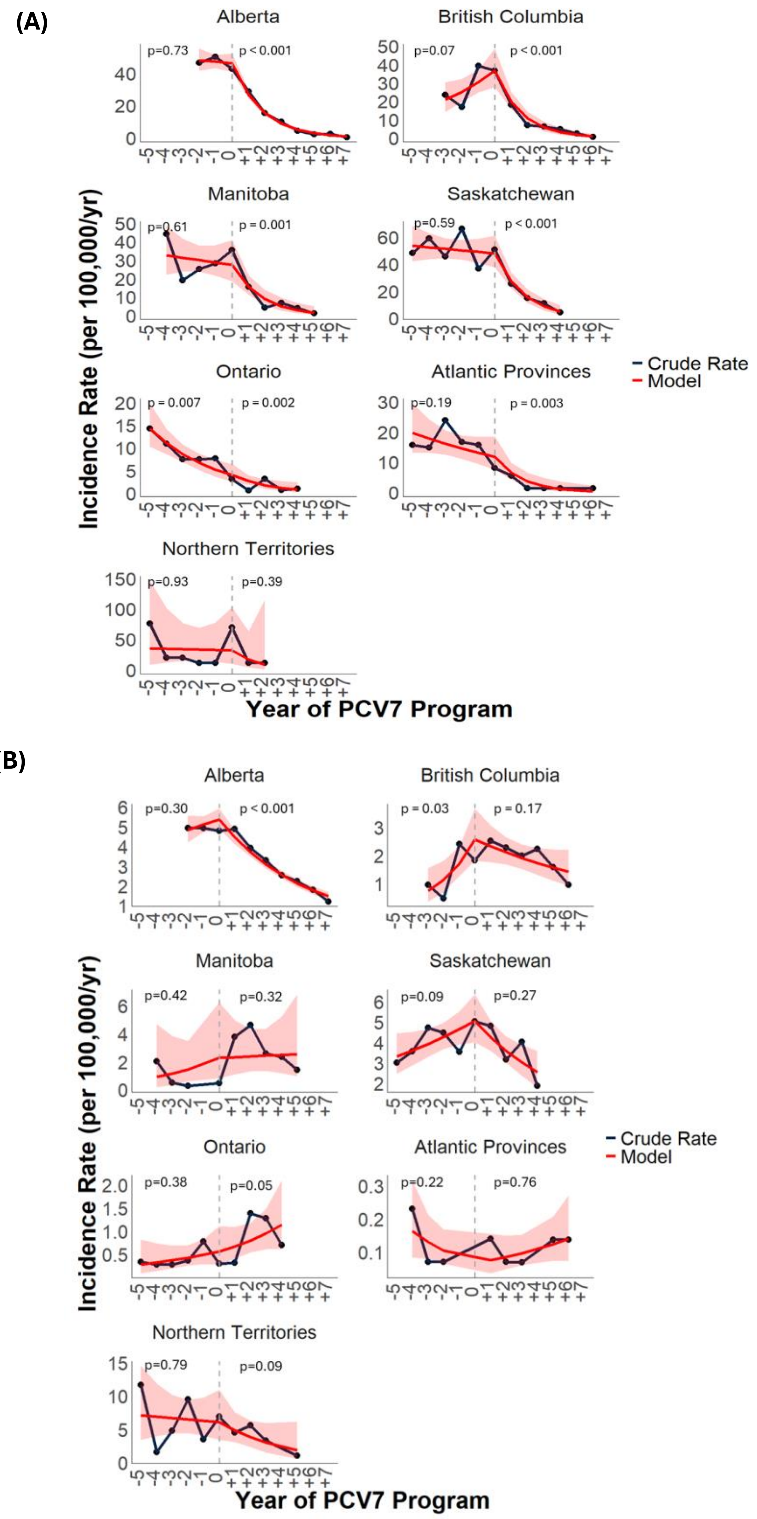

Supplementary Figure 4. Quasi-Poisson piecewise regression models showcasing the changes in incidence rates associated with the PCV7 program by region, where year 0 denotes the year of PCV7 introduction in A) the direct cohort (0-4 years old) and (B) the indirect cohort (≥ 10 years old).

© 2026 Ramos B et al. *JAMA Network Open*.

(A)

(B)

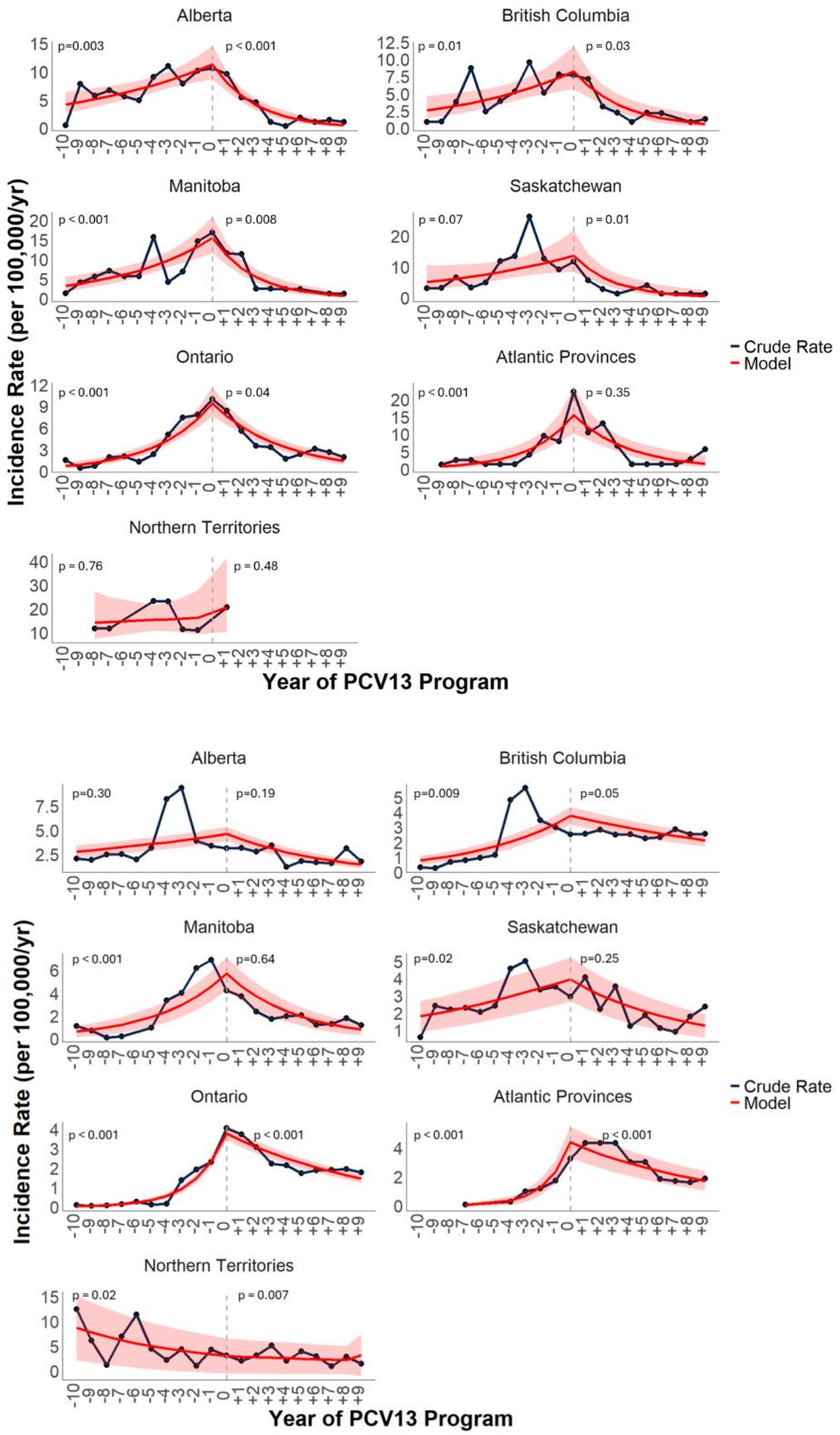

Supplementary Figure 5. Quasi-Poisson piecewise regression models showcasing the changes in incidence rates associated with the PCV13 program by region, where year 0 denotes the year of PCV13 introduction in A) the direct cohort (0-4 years old) and (B) the indirect cohort (≥ 10 years old).

© 2026 Ramos B et al. *JAMA Network Open*.

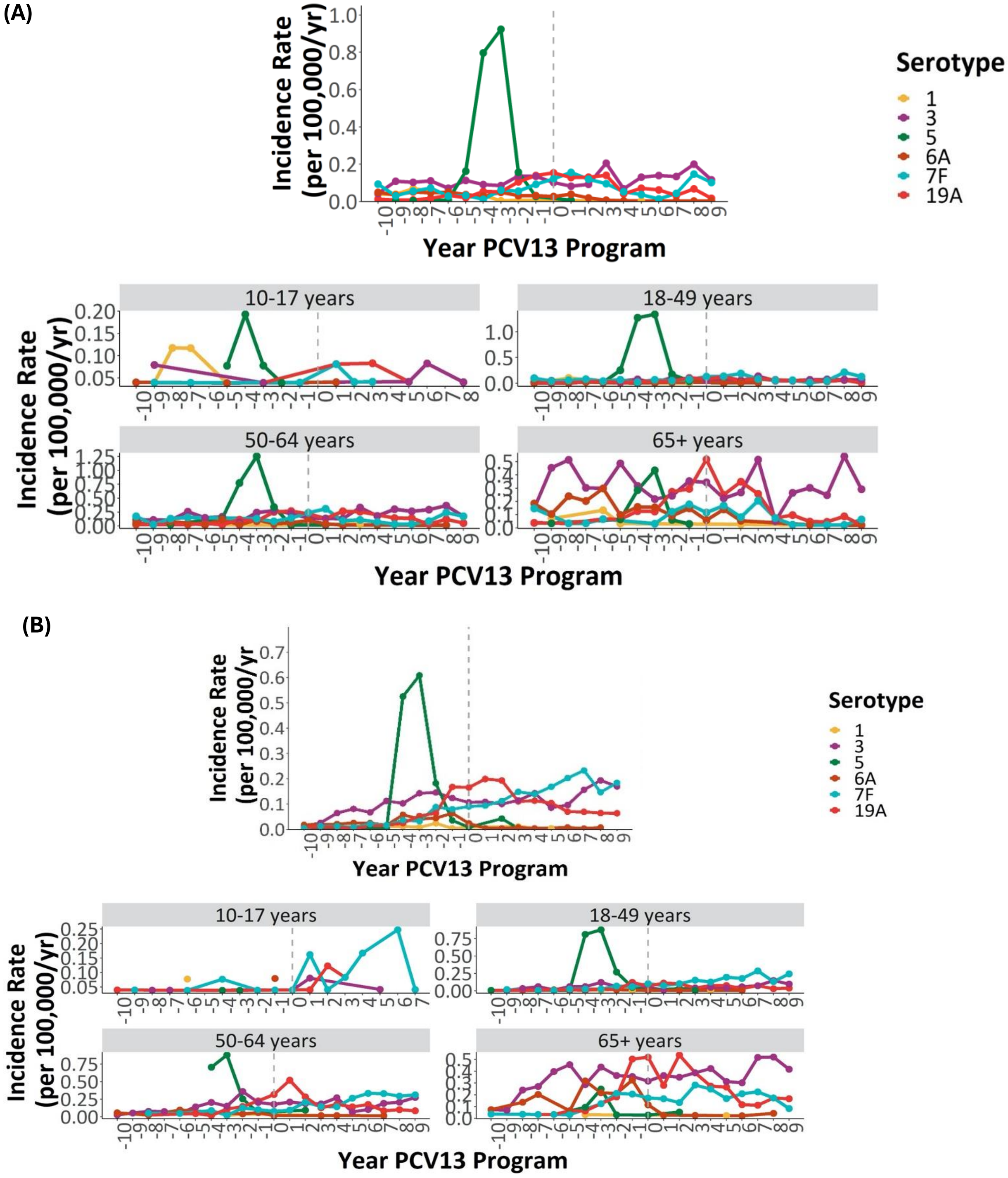

Supplementary Figure 6. Breakout of serotype 5 in (A) Alberta and (B) British Columbia from year -5 to year -3 during the observation of the PCV13 program in the indirect population and occurred in adults ≥18 years.

© 2026 Ramos B et al. *JAMA Network Open*.

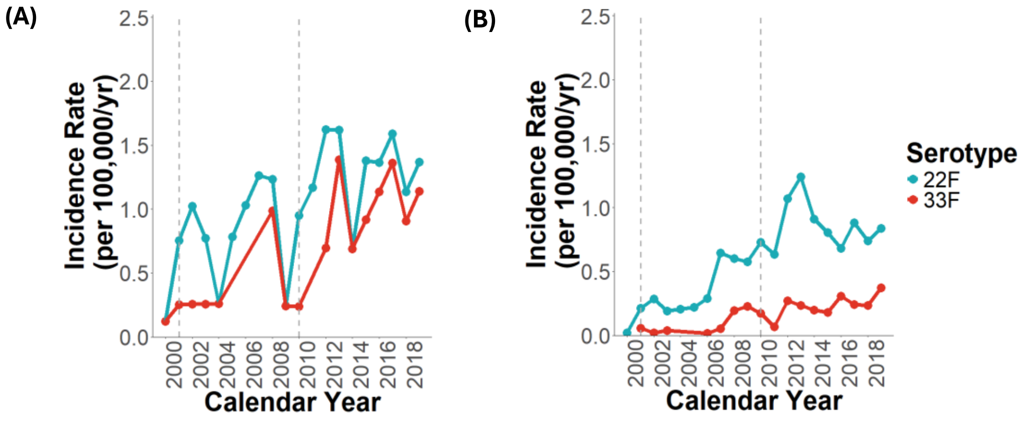

Supplementary Figure 7. Incidence rates of PCV15/non-PCV13 serotypes in the (A) direct cohort and (B) the indirect cohort.

© 2026 Ramos B et al. *JAMA Network Open*.

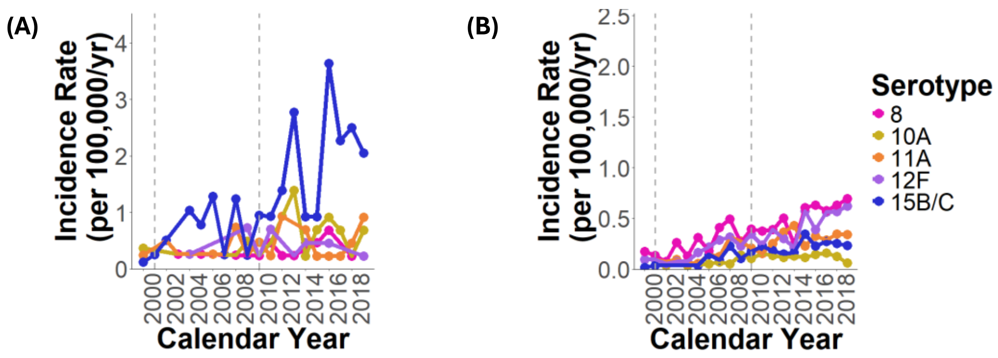

Supplementary Figure 8. Incidence rates of PCV20/non-PCV13 serotypes in the (A) direct cohort and (B) the indirect cohort.

© 2026 Ramos B et al. *JAMA Network Open*.

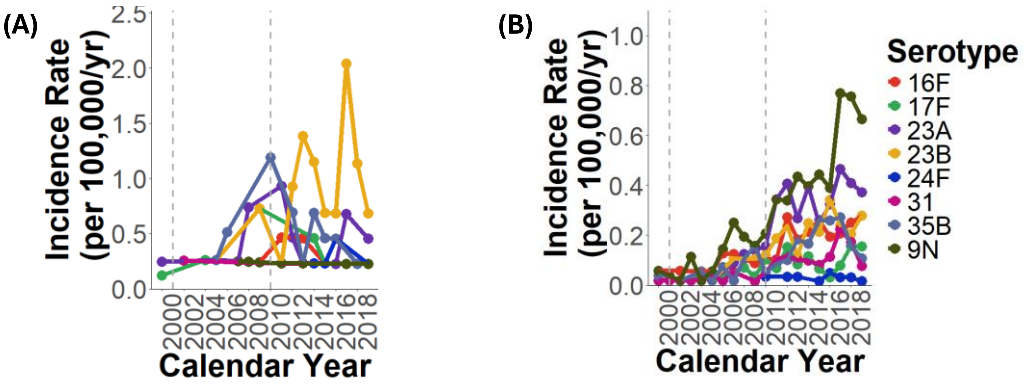

Supplementary Figure 9. Incidence rates of PCV21/non-PCV20 serotypes in the (A) direct cohort and (B) the indirect cohort. There were no cases of serotype 20A.

© 2026 Ramos B et al. *JAMA Network Open*.

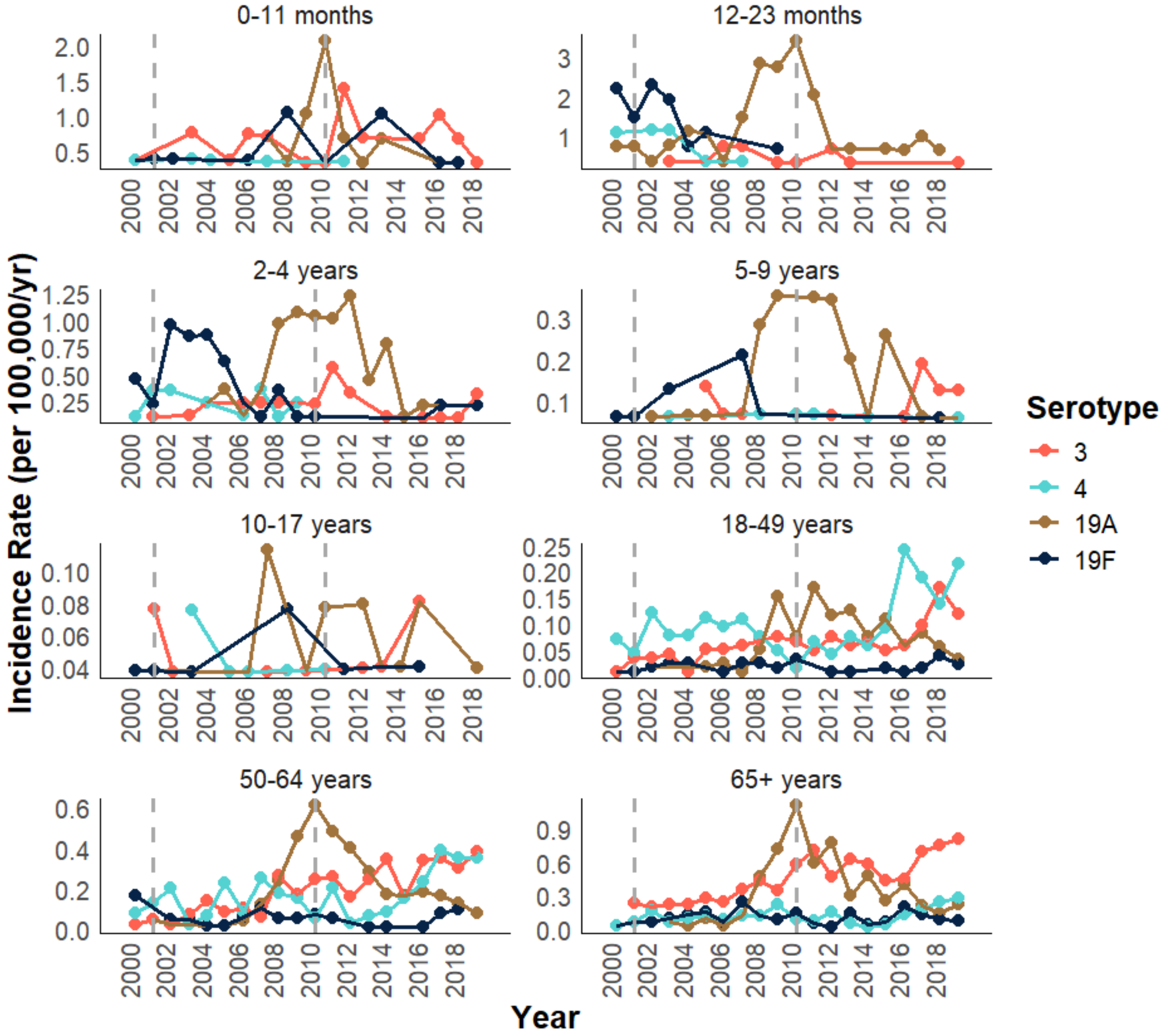

Supplementary Figure 10. Incidence rates of persistent serotypes 3, 4, 19A, and 19F by age group.

© 2026 Ramos B et al. *JAMA Network Open*.

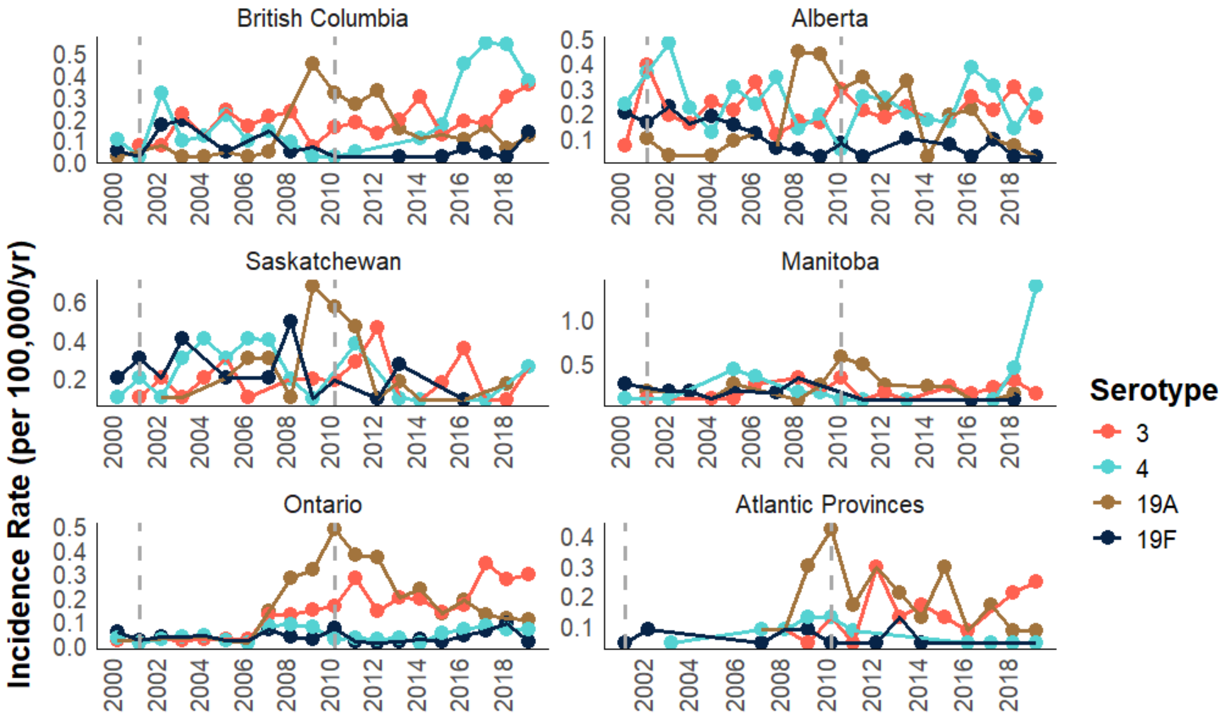

Supplementary Figure 11. Incidence rates of persistent serotypes 3, 4, 19A, and 19F by province. Incidence rates for the Northern Territories were excluded due to low number of isolates.

© 2026 Ramos B et al. *JAMA Network Open*.
