# Supplementary material for: Invasive Pneumococcal Disease Epidemiology and Conjugate Vaccines in Canada, 2000-2019
Source: JAMA Netw Open. 2026 Apr 9;9(4):e266005. doi: 10.1001/jamanetworkopen.2026.6005 (PMC13067013; doi:10.1001/jamanetworkopen.2026.6005)
Supplement: Supplement 2. — Data Sharing Statement [file jamanetwopen-e266005-s002.pdf]

Data Sharing Statement

Ramos. Invasive Pneumococcal Disease Epidemiology and Conjugate Vaccines in Canada, 2000-2019. *JAMA Netw Open*. Published April 09, 2026.
doi:10.1001/jamanetworkopen.2026.6005

Data
Data available: No
